# What Is the Relationship between Haptoglobin, Malaria, and Anaemia?

**DOI:** 10.1371/journal.pmed.0030200

**Published:** 2006-05-02

**Authors:** Stephen Rogerson

## Abstract

Rogerson discusses a new study that found that children with the haptoglobin (Hp) 2-2 genotype had a greater fall in hemoglobin over the malaria season than children with other Hp genotypes.

In malaria-endemic countries of Africa, anaemia is very common in pregnant women and in children under five. Although anaemia is multifactorial—causative factors include iron deficiency and other nutritional deficiencies, helminth infection, and HIV—malaria is clearly an extremely important factor. Over half of malaria-related deaths are attributed to severe malaria anaemia (which is defined as malaria parasitaemia and a haemoglobin (Hb) concentration less than 50 g/l) [1]. Several antimalarial interventions have been shown to prevent anaemia, including insecticide-treated nets, residual spraying, malaria chemoprophylaxis, and, more recently, intermittent presumptive treatment of infants (i. e., antimalarials coadministered with childhood immunization). Insecticide-treated nets have been shown to decrease all-cause mortality [2].

The pathogenesis of malaria anaemia remains incompletely understood. Dyserythropoiesis (disordered red cell development, which is, at least in part, due to inflammatory cytokines acting on erythroid precursors), intravascular haemolysis of infected red cells, and destruction of both parasitized and uninfected erythrocytes by splenic macrophages are all important [3,4]. Interestingly, it has been estimated that ten or more uninfected erythrocytes may be lost for each infected one [5], presumably because malaria infection alters uninfected erythrocytes. The probable causes of red cell loss include oxidation of band 3 (the anion transporter of the erythrocyte membrane) or membrane lipids, and deposition of IgG, complement, or immune complexes on the erythrocyte surface.

## The Possible Role of Haptoglobin in Malaria Anaemia

The glycoprotein haptoglobin (Hp) is the body's main tool for removing circulating, toxic free Hb during intravascular haemolysis. Hp binds rapidly and with high affinity to free Hb, with the resultant complex being taken up by CD163, which is expressed on monocytes/macrophage lineage cells. Binding of Hp/Hb complexes to CD163 leads to cytokine secretion by macrophages [6]. In the absence of Hp, free Hb can enter and damage renal glomeruli, and is a potent oxidant that can lead to the generation of reactive oxygen species.

The pathogenesis of malaria anaemia remains incompletely understood.

In humans, unlike other animals, Hp exists in two forms. The *Hp^2^* gene arose by duplication of a region of the gene encoding the α chain of *Hp^1^*, leading to three phenotypes—1-1, 2-1, and 2-2. Hp1-1 leads to smaller, more abundant dimers with a higher affinity for Hb, and to Hp/Hb complexes with a lower affinity for CD163. Studies have associated the Hp2-2 phenotype with increased morbidity from cardiovascular disease, diabetes, HIV infection, and other conditions—perhaps because concentrations of Hp2-2 are lower, Hp2-2 is less able than Hp1-1 to penetrate extravascular spaces, and Hp2-2 is less able to prevent oxidative damage or immune activation than the other phenotypes [7].

Given the importance of intravascular haemolysis in malaria infection, studies of Hp in malaria are important, but results to date have been inconclusive. Earlier studies based on phenotyping suggested Hp1-1 was associated with severe or symptomatic infection [8,9]. For example, in one study of 72 patients in Sudan with cerebral malaria, the percentage of patients with Hp phenotypes 1-1, 2-1, and 2-2 were 63.9%, 29.2%, and 6.9%, respectively [8]. But the low prevalence of Hp2-2 and the high prevalence of Hp1-1 in the patients in this study may reflect lack of sensitivity of the electrophoretic technique used in the study to detect the less abundant (and multiple) bands of Hp2-2 rather than the single abundant Hp1-1. Circulating levels of Hp1-1 are normally significantly higher than those of Hp2-2, but levels fall during malaria infection due to clearance of Hp/Hb complexes; age may influence the degree of fall in Hp with malaria [10]. More recently, PCR-based genotyping studies have examined associations between *Hp* genotype and severe malaria or associations between *Hp* genotype and mild disease [11,12]. These studies did not find clear associations with *Hp* genotype; although, in one study, there was a suggestion of an association with severe malaria, especially severe anaemia.

## A New Study

In a new study in *PLoS Medicine*, Atkinson and colleagues have taken a different approach [13]. Working in The Gambia, where malaria transmission is intense but highly seasonal and anaemia is a common complication, they followed a group of children over the malaria season. The authors used Hb concentration as their main readout. Although baseline Hb levels were similar across *Hp* genotypes, children who were *Hp^2/2^* had a greater fall in Hb (by 4 g/l) over the malaria season than did other children. *Hp* genotype significantly predicted Hb levels at the end of the malaria season in multivariate analysis, together with iron status and several other variables. A separate analysis examining factors influencing the magnitude of the fall in Hb gave similar results. Additionally, children with *Hp^2/2^* genotype were more likely to be parasitaemic. The authors suggest that the effect of *Hp* genotype on the fall in Hb (and, thus, possible protection from anaemia) may be more significant than that of sickle cell trait (HbAS) or glucose-6-phosphate dehydrogenase deficiency. On the other hand, sickle cell trait and glucose-6-phosphate dehydrogenase deficiency have been more compellingly associated with protection from severe malaria than have *Hp* genotypes [14].

## How Does Hp Affect Hb Levels?

How does *Hp* genotype influence Hb fall? The authors offer several interesting suggestions. Because destruction of uninfected erythrocytes may be especially important in development of malaria anaemia, this mechanism is one plausible route. Oxidative damage to uninfected cells might be more marked in Hp2-2 individuals since Hp2-2 proteins bind less efficiently to Hb, and since levels of Hp2-2 are lower and more easily depleted, increasing premature destruction of erythrocytes. Another reason *Hp* genotype may influence Hb levels might be because complexes of Hp2-2 with Hb, but not other Hp/Hb complexes, enter monocytes (which express less CD163 than macrophages), stimulating cytokine release by these circulating cells. Thus, different Hp types inducing different cytokine responses may affect the duration of inflammatory, marrow-suppressive cytokine production following malaria. Persistent marrow suppression, as judged by inappropriately low reticulocyte responses after resolution of infection, is a feature of malaria [3,4]. And *Hp^2/2^* genotype has been associated with increased iron accumulation in haemochromatosis—in one study but not in others (reviewed in [7])—suggesting *Hp* genotype may have some role in iron homeostasis. Interestingly, the 17% prevalence of *Hp^2/2^* in Atkinson and colleagues' study was much lower than that which was reported in European populations [7], suggesting a possible protective role for *Hp^1^* genotypes against malaria.

## Public Health Implications

Is a 4 g/l difference in Hb fall clinically relevant? Probably. Although the effects of this fall on the proportion of children suffering moderate or severe anaemia are not given in the study, the population distribution of Hb levels is wide. A fall in mean Hb may translate into increased numbers of individuals with symptomatic disease, although this effect is not demonstrated in this study. From a public health perspective, interventions that result in a rise in Hb of similar magnitude—such as intermittent presumptive treatment in pregnancy [15]—are endorsed by the World Health Organization. And small changes in group levels may hide subgroups with more dramatic changes in Hb. How one might identify individuals at risk for the more dramatic falls in Hb, or whether antimalarial measures would prevent such falls, are unresolved questions.

## Abbreviations

Hb: haemoglobin
Hp: haptoglobin
